# A case series of patients with cardiac amyloidosis evaluated at a Colombian university hospital

**DOI:** 10.3389/fcvm.2025.1487717

**Published:** 2025-02-03

**Authors:** Juan David López-Ponce de León, Santiago Granados-Álvarez, Juan Pablo Arango-Ibanez, Juan Manuel Montero Echeverri, Andrea Alejandra Arteaga Tobar, Andrea Facio-Lince Garcia, Yorlany Rodas Cortes, Juan Esteban Gómez-Mesa

**Affiliations:** ^1^ Centro de Investigaciones Clínicas, Fundación Valle del Lili, Cali, Colombia; ^2^ Departamento de Cardiología, Fundación Valle del Lili, Cali, Colombia; ^3^ Facultad de Ciencias de la Salud, Universidad Icesi, Cali, Colombia

**Keywords:** amyloidosis, AL amyloidosis, cardiac amyloidosis, Colombia, Latin America, transthyretin

## Abstract

**Background:**

In Colombia, the characteristics of cardiac amyloidosis (CA)—including wild-type transthyretin amyloidosis (ATTRwt), immunoglobulin light chain amyloidosis (AL), and genetic variant transthyretin amyloidosis (ATTRv)—are underexplored.

**Methods:**

This case series at a Colombian university hospital analyzed demographic, clinical, laboratory, radiological, and genetic data of CA patients diagnosed between 2018 and 2022. Patients with incomplete data underwent further testing.

**Results:**

Of 24 identified patients, 14 were included after exclusions. The majority were male (73.3%), with an average age of 70.6 years. ATTRv and AL were equally prevalent (42.8%), followed by ATTRwt (14.2%). The p.Val142Ile TTR mutation was found among all ATTRv patients. Most presented with functional capacity NYHA I-II and common electrocardiographic findings included low voltage, atrial fibrillation, and first-degree AV block. Echocardiography and cardiac magnetic resonance imaging revealed ventricular hypertrophy, diastolic dysfunction, reduced longitudinal strain, and late myocardial enhancement.

**Conclusions:**

AL and ATTRv were the most common causes of CA followed by ATTRwt. This distribution, along with the clinical, and radiological characterization is consistent with previous reports of other regions. The p.Val142Ile mutation was the only one found in patients with ATTRv, suggesting a strong African genetic influence. These findings enhance our understanding of CA in the region.

## 1 Introduction

Amyloidosis constitutes a rare spectrum of disorders characterized by the extracellular accumulation of amyloid fibrils. These fibrils are insoluble and misfolded aggregates of low molecular weight protein that frequently deposit in various organs, including the kidneys, skin, and heart, among others. Cardiac amyloidosis (CA) occurs when amyloid deposits in the heart, leading to thickening and stiffening of ventricular walls and generating heart failure, conductive dysfunction, and restrictive cardiomyopathy (1). The most common forms of CA include light-chain related amyloidosis (AL) with an incidence of 12 cases per million persons/year, wild-type transthyretin-related amyloidosis (ATTRwt) with a prevalence of 141 cases per million persons/year, and hereditary transthyretin amyloidosis (ATTRv, also known as ATTRmt), with an incidence of 0.3 cases per million persons/year (2). Both ATTRwt and ATTRv lead to cardiac amyloidosis through transthyretin deposition (ATTR-CA). These three types of CA constitute 98% of cases (1, 3).

In AL amyloidosis, amyloid formation is driven by an excess of immunoglobulin light chains, often due to a primary monoclonal gammopathy or as part of other hematological conditions. In ATTRwt, amyloid deposits form due to the instability of the transthyretin protein associated with aging, without the influence of genetic mutations. Conversely, ATTRv results from point mutations in the transthyretin gene—most commonly p.Val142Ile (formerly p.Val122Ile), p.Val30Met, and p.Thr60Ala. These mutations lead to the production of an unstable protein that is prone to misfolding and subsequent amyloid deposition (1, 2).

The main pathological consequences of CA include thickening and fibrosis of the interstitium classically causing restrictive cardiomyopathy and other complications such as atrial fibrillation (AF), valvular heart disease, conduction system dysfunction, ventricular arrhythmias, and coronary heart disease (1, 2). Certain laboratory, electrocardiographic, radiological, and echocardiographic findings serve as “red flags” to suspect CA, and these include disproportionally elevated N-terminal pro-B-type natriuretic peptide (NT-proBNP), persistently positive troponins, low voltage or pseudoinfarct patterns, hypertrophy with no dilation, myocardial speckle, valvular thickening, and reduced strain with apical sparing (3). Other extracardiac findings are also frequently seen and should be sought to increase the suspicion of the disease (3, 4).

The clinical, laboratory, and radiological characteristics of CA are underexplored in Latin America (LATAM), especially in Colombia, as are the mutations underlying ATTRv. Moreover, a lack of awareness of CA among clinicians has been reported in LATAM (5). Our study aims to contribute to the knowledge of this disease in the region by characterizing cases of CA evaluated in a high-complexity referral university hospital in Colombia. This is essential, given the increasing worldwide recognition and prevalence of CA due to improved awareness and higher cardiac imaging quality, especially in patients with heart failure (6, 7). New research on CA in LATAM is crucial to address significant gaps in clinician awareness and diagnostic practices, enhancing early detection and management of this disease (5).

## 2 Materials and methods

### 2.1 Design and participants

This is a case series of patients with confirmed CA evaluated at a university hospital in Cali, Colombia between 2018 and 2022. Patients were included if CA was confirmed. Exclusion criteria included patients whose CA diagnosis was subsequently ruled out, and those with insufficient data for a definitive diagnosis of CA who could not be reached for further testing. The diagnosis of CA was established following the 2021 guidelines outlined in the European Society of Cardiology (ESC) position statement on myocardial and pericardial diseases (3). Diagnostic criteria for CA according to the ESC are summarized in Table 1.

**Table 1:** Diagnostic criteria of cardiac amyloidosis simplified from the European Society of Cardiology position statement on myocardial and pericardial diseases (3).

Diagnosis of AL cardiac amyloidosis (all):
Positive monoclonal peaks on serum electrophoresis Positive bone marrow biopsy or abdominal fat biopsy Echocardiography or magnetic resonance with typical features of cardiac amyloidosis
Diagnosis of ATTRwt cardiac amyloidosis (all):
Negative monoclonal peaks on serum electrophoresis Bone scintigraphy PYP-99mTc grade 2–3 OR grade 1 with positive cardiac/extracardiac biopsy Genetic testing showing wild-type *TTR* alleles
Diagnosis of ATTRv cardiac amyloidosis (all):
Negative monoclonal peaks on serum electrophoresis Bone scintigraphy PYP-99mTc grade 2–3 or grade 1 with positive cardiac biopsy Genetic testing showing *TTR* pathogenic variant

### 2.2 Data collection

The primary objective of this study was to gather comprehensive demographic, clinical, laboratory, and radiological data of patients. Data collection was primarily conducted through the institutional medical record system. In instances where the available data did not indicate CA conclusively, yet a high level of suspicion existed, participants were contacted and invited to join the study. Diagnostic procedures, including echocardiography, contrast-enhanced cardiac magnetic resonance imaging (CMRI), pyrophosphate technetium-99 m scintigraphy (PYP-99mTc), and genetic testing were employed to establish a definitive diagnosis. At our institution, the interpretation of these radiological tests in the context of CA is performed by either an experienced cardiologist or a radiologist with a fellowship in cardiovascular imaging. Genetic sequencing of exons 2, 3, and 4 was performed using blood samples to assess pathogenic variants of the TTR gene. Research Electronic Data Capture (REDCap) platform (version 14.7.5, © 2024 Vanderbilt University) was used for data storage. The most recent results available were utilized for this study for clinical, laboratory, and imaging data. The integrity and accuracy of the data were evaluated by two researchers using a random 33% sample from the database. Furthermore, a comprehensive assessment was conducted to identify outliers and inconsistencies across the entire database.

### 2.3 Definitions

Positive troponin was considered when values were greater than the 99th percentile using PATHFAST TM hs-cTnI. Left ventricular (LV) hypertrophy was considered if the LV mass was greater than 115 g/m^2^ for men and 95 g/m^2^ for women, as previously defined by the American Society of Echocardiography (ASE) and the European Association of Echocardiography (EAE) (8). Furthermore, diastolic dysfunction was defined following recommendations of the ASE and EAE (9). Reduced longitudinal LV strain was considered when less than −15% (3).

### 2.4 Statistical analysis

Descriptive statistics were applied to the collected data. Continuous variables are summarized with mean and standard deviation (SD) for normally distributed data, and median and interquartile range (IQR) for non-normally distributed data. Qualitative data is presented in terms of absolute frequencies and percentages. These percentages reflect the proportion of the total available data (in case of missing data). The Shapiro-Wilk test was used to evaluate distribution. All descriptive analyses were conducted using RStudio, version 2023.12.0 + 369.

### 2.5 Ethical considerations

This study was approved by the Comité de Ética e Investigación Biomédica (number 2022.1913), which is the institutional review board at Fundación Valle del Lili. All patients who were contacted for complementary studies signed an informed consent. This study is in line with the Declaration of Helsinki.

## 3 Results

**Figure 1:**
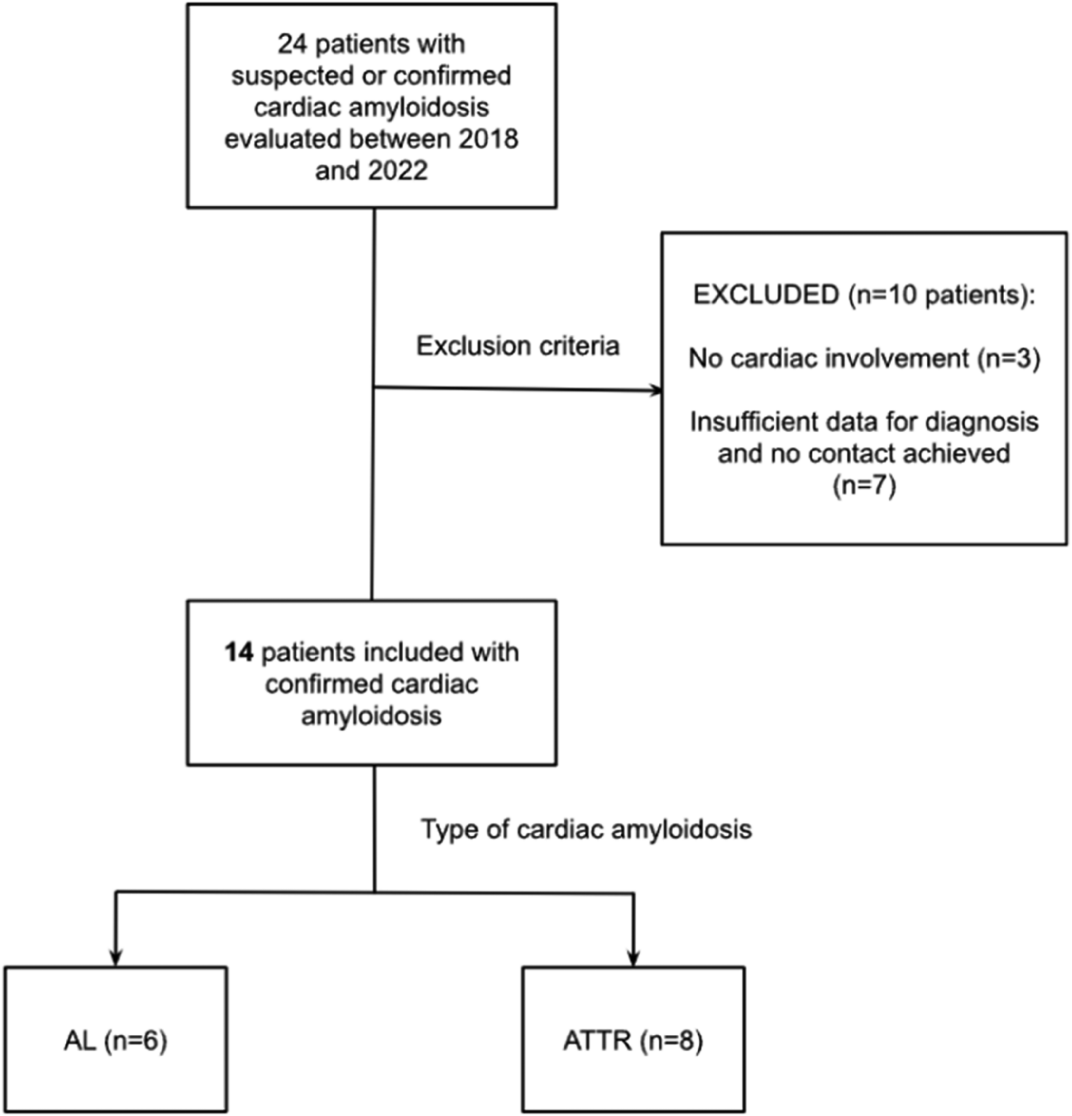
Flowchart of patient selection. AL, Light Chain amyloidosis; ATTR, Transthyretin amyloidosis.

A total of 24 patients with suspected or confirmed CA were evaluated between 2018 and 2022 at our institution. We excluded three patients with amyloidosis without features of cardiac amyloidosis. Two of these patients had a pathogenic TTR variant (p.Val142Ile) and one had AL amyloidosis with elevated cardiac biomarkers. Seven patients were excluded due to insufficient clinical data to confirm or discard the diagnosis. For those patients, no answer was obtained despite repeated contact attempts to complete the required information. Finally, 14 patients were included, 6 (42.8%) with AL amyloidosis and 8 (57.2%) with ATTR (6 with ATTRv and 2 with ATTRwt) (Figure 1). The pathogenic variant p.Val142Ile of the *TTR* gene was found in all cases of the ATTRv. Importantly, genetic testing was conducted on all participants in the study to explore the potential for multiple underlying causes of CA within the same individual. No pathogenic variants were detected in patients with AL amyloidosis.

The mean age at evaluation was 70.6 (SD ±11.5) years, being higher for ATTR (75.6 ± 8.4) than for AL (64 ± 12.3). All patients with ATTR were male and 50% of patients in the AL group were men. More patients in the AL group had hypertension and chronic kidney disease. In the ATTR group a higher proportion of patients presented with New York Heart Association Functional Classification (NYHA) III or IV and higher NT-proBNP. Other clinical and laboratory data are summarized in Table 2. Neurological involvement, defined as the presence of neuropathy, was observed in 2 (33.3%) patients with AL amyloidosis. Musculoskeletal involvement, defined by any tendon rupture, carpal tunnel syndrome, or spinal stenosis, was observed in 7 (50%) patients, 5 (62.5%) with ATTR. Renal involvement, defined by a glomerular filtration rate less than 60 ml/min calculated with CKD-EPI, was found in 8 (57.1%) patients, 4 (66.6%) with AL amyloidosis, and 4 (50%) with ATTR amyloidosis.

**Table 2:** Demographics, comorbidities, and laboratories.

Variable	Total, *n* = 14	AL, *n* = 6	ATTR, *n* = 8
Age at evaluation, mean (SD)	70.6 (11.5)	64 (12.3)	76.6 (8.4)
Male sex, *n* (%)	11 (78.5%)	3 (50%)	8 (100.0%)
Age at diagnosis, mean (SD)	67.4 (12.2)	58.6 (11.6)	74 (8.7)
Hypertension, *n* (%)	9 (64.2%)	5 (83.3%)	4 (50%)
Hypotension, *n* (%)	3 (21.4%)	2 (33.3%)	1 (12.5%)
Diabetes mellitus, *n* (%)	2 (14.2%)	1 (16.6%)	1 (12.5%)
Lung disease, *n* (%)	1 (7.1%)	1 (16.6%)	0 (0%)
Coronary artery disease, *n* (%)	5 (35.7%)	2 (33.3%)	3 (37.5%)
Chronic kidney disease, *n* (%)	8 (57.1%)	4 (66.6%)	4 (50%)
Carpal tunnel syndrome, *n* (%)	4 (28.5%)	2 (33.3%)	2 (25%)
Spinal stenosis, *n* (%)	1 (7.1%)	1 (16.6%)	0 (0%)
Biceps tendon rupture, *n* (%)	3 (21.4%)	0 (0%)	3 (37.5%)
NYHA I-II, *n* (%)	10 (71.4%)	6 (100%)	4 (50%)
NYHA III-IV, *n* (%)	4 (28.6%)	0 (0%)	4 (50%)
NT-proBNP^a^, pg/ml, median (IQR)	902 (465, 4,776)	766 (278, 902)	3,844 (820.2, 9,923.2)
Positive troponin^b^, *n* (%)	6 (60%)	3 (60%)	3 (60%)

AL, Light Chain amyloidosis; ATTR, Transthyretin amyloidosis; IQR, interquartile range; NYHA, New York Heart Association Functional Classification; NT-proBNP, N-terminal prohormone of brain natriuretic peptide; SD, Standard deviation.

^a^ Missing data—Total: 3; AL: 1; ATTR: 2.

^b^ Missing data—Total: 4; AL: 1; ATTR: 3.

The most common electrocardiographic findings were low voltage, AF, and first-degree atrioventricular block (*n* = 4, 30.8% each) (Table 3).

**Table 3:** Electrocardiographic findings.

Finding	Total^a^, *n* (%)	AL, *n* (%)	ATTR, *n* (%)
Low voltage	4 (30.8%)	1 (20%)	3 (37.5%)
Atrial fibrillation	4 (30.8%)	1 (20%)	3 (37.5%)
Right bundle branch block	1 (7.7%)	0 (0%)	1 (12.5%)
Left bundle branch block	2 (15.4%)	0 (0%)	2 (25.0%)
Pseudo-infarct pattern	3 (23.1%)	1 (20%)	2 (25%)
First-degree atrioventricular block	4 (30.8%)	1 (20%)	3 (37.5%)

AL, Light Chain amyloidosis; ATTR, Transthyretin amyloidosis.

^a^ Missing data—Total: 1; AL: 1.

Transthoracic echocardiography demonstrated reduced left-ventricular ejection fraction (LVEF) in 62.5% of patients with ATTR but in none of the AL patients. The LV wall and septum thickness were higher for ATTR individuals (15.3 ± 2.9 and 14 IQR 12.5–19 vs. 13 ± 2.6 and 13.5 IQR 12.2–14), as was the mass index (164.7 ± 31.2 vs. 119.6 ± 33.9). Diastolic dysfunction and reduced longitudinal strain were present in most patients. Other echocardiographic findings are described in Table 4. The median time in months between diagnosis and echocardiography was 265.5 (125.5, 648) days. The echocardiography with the most complete assessment regarding the variables used in this study was chosen.

**Table 4:** Findings on transthoracic echocardiography.

Finding	Total	AL	ATTR
Left ventricular diastolic diameter^a^, mm, mean (SD)	44.3 (6.8)	42.1 (4.8)	46.3 (8)
Left ventricular systolic diameter^a^, mm, mean (SD)	34.2 (7)	31.6 (6.4)	36.4 (7.3)
Ejection fraction,%, mean (SD)	48.1 (15.6)	63 (4.4)	37 (11)
Ejection fraction <40%, *n* (%)	5 (35.7%)	0 (0%)	5 (62.5%)
Left ventricular wall thickness^a^, mm, mean (SD)	14.2 (2.9)	13 (2.6)	15.3 (2.9)
Septum thickness^a^, mm, median (IQR)	14 (12, 14)	13.5 (12.2, 14)	14 (12.5, 19)
Left ventricular mass index^b^, g/m^2^, mean (SD)	145.9 (38.6)	119.6 (33.9)	164.7 (31.2)
Left ventricular hypertrophy^a^, *n* (%)	12 (92.3%)	5 (83.3%)	7 (100%)
Symmetric hypertrophy, *n* (%)	11 (91.6%)	6 (100%)	6 (85.7%)
Diastolic dysfunction^c^, *n* (%)	4 (30.7%)	2 (33.3%)	5 (83.3%)
Speckled myocardium^a^, *n* (%)	−11.4 (2.8)	−13 (2.1)	2 (28.6%)
Longitudinal strain^d^,%, mean (SD)	11 (100%)	6 (100%)	−9.7 (2.6)
Reduced strain^d^, *n* (%)	3 (21.4%)	1 (16.6%)	5 (100%)
Thickened mitral valves^a^, *n* (%)	38.5 (16.3)	37.6 (17.5)	2 (28.6%)
Pulmonary artery systolic pressure, mmHg^e^, mean (SD)	2 (16.6%)	1 (16.6%)	39.1 (16.8)
Systolic anterior motion of the mitral valve^f^, *n* (%)	44.3 (6.8)	42.1 (4.8)	1 (16.7%)

AL, Light Chain amyloidosis; ATTR, Transthyretin amyloidosis; IQR, Interquartile range; SD, Standard deviation.

^a^ Missing data—Total: 1; ATTR: 1.

^b^ Missing data—Total: 2; AL: 1; ATTR: 1.

^c^ Missing data—Total: 2; ATTR: 2.

^d^ Missing data—Total: 3; ATTR: 3.

^e^ Missing data—Total: 1; AL: 1.

^f^ Missing data—Total: 2; ATTR: 2.

CMRI identified a thickened septum in 86.6% of patients with this imaging technique, the mean left atrial area and mass index were higher for ATTR patients. All patients with CMRI had late contrast enhancement, abnormal T1 mapping, an elevated extracellular volume. These findings are shown in Table 5. The median time in months between diagnosis and cardiac magnetic resonance was 108 (−22, 527) days. If many CMRIs were available in a patient, the one closest to the diagnosis was chosen. One patient with ATTR-CA had no available CMRI (Table 5).

**Table 5:** Findings on magnetic resonance with contrast.

Finding	Total	AL	ATTR
Septum thickness^a^, mm, mean (SD)	14.9 (3.3)	14.1 (2.6)	15.6 (4)
Septum >12 mm^a^, *n* (%)	11 (84.6)	5 (83.3%)	6 (85.7%)
Posterior wall thickness^a^, mm, mean (SD)	15.3 (3.4)	15 (3.1)	15.6 (4)
Left atrial area^b^, cm^2^, mean (SD)	60.8 (21.4)	55.8 (30.7)	64.9 (11)
Mass index^a^, g/m^2^, mean (SD)	110.7 (4.5)	107.3 (52.8)	113.7 (33.1)
Late gadolinium enhancement^a^, *n* (%)	13 (100%)	6 (100%)	7 (100%)
Extracellular volume >25%^c^, *n* (%)	10 (100%)	4 (100%)	6 (100%)
Elevated T1 mapping^c^, *n* (%)	10 (100%)	4 (100%)	6 (100%)

AL, Light Chain Amyloidosis; ATTR, Transthyretin amyloidosis; SD, Standard deviation.

^a^ Missing data—Total: 1; ATTR: 1.

^b^ Missing data—Total: 3; AL: 1; ATTR: 2.

^c^ Missing data—Total: 4; AL: 2; ATTR: 2.

99m-Tc-PYP was obtained from all patients. 87.5% of ATTR patients had Perugini scores of 2 or 3, and one patient (12.5%) had a Perugini score of 1 (diagnosis confirmed through endomyocardial biopsy). In the AL group, 4 (66.6%) patients had a Perugini score of 0, and 2 (33.3%) had a score of 1.

In the Supplementary material, we present imaging findings of patients from our series (Figures S2–S4 of the Supplementary material).

At the time of recruitment, 13 patients (92.8%) were receiving guideline-directed medical therapy for heart failure. The use of specific medications among these patients was as follows: beta-blockers in 9 patients (64.2%), aldosterone receptor antagonists in 11 (78.5%), angiotensin-converting enzyme inhibitors in 1 (7.1%), angiotensin receptor blockers in 3 (21.4%), sodium-glucose cotransporter-2 inhibitors in 8 (57.1%), and sacubitril in 1 (7.1%). Loop diuretics were prescribed to 11 patients (78.5%), 4 (66.6%) with AL amyloidosis, and 7 (78.5%) with ATTR. Of the ATTR amyloidosis group, 2 patients (25%) were treated with tafamidis. Management of AL amyloidosis included bone marrow transplant in 50% of patients and chemotherapy in 83.3%.

By January 1st, 2024 all patients included in this study were alive according to their affiliation status in Administrator of the Resources of the General Health Social Security System of the Colombian Government.

## 4 Discussion

This study aimed to characterize patients with CA evaluated in a single center in Colombia, between 2018 and 2022. Among 14 patients included, we found that the most common causes of CA were AL and ATTRv. These findings are consistent with previous reports that demonstrate these as the most common causes. An Italian study by Porcari et al, with 182 patients with CA, reported a prevalence of 47.3% for AL CA and 44.5% for ATTR-CA (10), and a Spanish study by Barge-Caballero et al, with 105 patients with CA, found that 62% were attributed to ATTR and 38% to AL (11). The same author conducted a study including 143 patients assessed between 2018 and 2020 and found a prevalence of 89.5% of ATTR and 10.5% of AL (12). López-Sainz et al. described a series of 180 Spanish patients, in which 64% had ATTR-CA and 36% had AL CA. The most common genetic variants found in this cohort were p.Val50Met and p.Val142Ile, representing two-thirds of patients with ATTRv (13).

Currently, there are few studies reporting case series of CA in South America. A study from Brazil included 105 patients with systemic amyloidosis, of which 83 were due to ATTR and 22 were due to AL. 68.7% of patients in the ATTR group were caused by pathogenic variants (ATTRv), the most common were pVale142Ile (45.6%) and p.Val50Met (40.3%). A cardiac phenotype was seen in 77.9% of patients with ATTR and in 90.9% of those with AL (14). A study from Argentina reported 167 cases of amyloidosis, of which 105 had cardiac involvement; of these, 52% were due to AL, 6% to ATTRv, 38% to ATTRwt, and 4% to serum amyloid A amyloidosis (15). A study from Peru included 8 patients with CA; 3 cases were due to ATTR, 3 by AL, and 2 had an unknown cause of CA (16).

To this date, more than 100 TTR gene mutations have been reported in the literature. The pathogenic variant seen in our cohort of patients is among the most common pathogenic variants worldwide (p.Val142Ile, p.Val30Met, and p.Thr60Ala) (7). Recognizing the variants that lead to ATTR-CA is crucial, as the clinical presentation may differ depending on the specific mutation (17, 18). The p.Val142Ile variant commonly damages the cardiovascular system and spares the conduction system commonly causing late-onset cardiomyopathy (18). Moreover, this variant is associated with worse quality of life and lower adjusted survival compared to other variants causing ATTRv. As seen in the ATTR cases of this study (mostly ATTRv by p.Val142Ile), there was a significant prevalence of electrocardiographic abnormalities and higher NYHA classifications, which are consistent with the previously described findings in the literature. Studies have demonstrated that the p.Val142Ile mutation is more prevalent in patients of African and Hispanic/Latino ancestry (18, 19). This genetic pattern could explain the high prevalence of this mutation in our study, given the considerable African ancestry in Colombia. Lastly, differentiation between ATTRv and ATTRwt is also clinically relevant, given the implications for genetic counseling and family screening in patients with ATTRv (7).

The median age at diagnosis of CA commonly ranges between the eighth and ninth decades, as recently reported by a meta-analysis (20), which is similar to our data. We found a male-predominant distribution in ATTR-CA (100%), which is consistent with previous evidence suggesting a higher risk of ATTR-CA among men (21). This distribution is not commonly evidenced in AL CA (22). The Spanish experience shows a similar age at diagnosis, 74.4 years, and a similar predominance of male patients (12). López-Sainz et al. showed a preponderance of male sex (64.1%) and a similar age at diagnosis (74.3 ± 12.7), their severity as measured by NYHA III or IV was similar to our series as were the extracardiac compromise (carpal tunnel syndrome, spinal stenosis, chronic kidney disease) (13). Interestingly, a third of our cohort had coronary artery disease, a finding described in only 6.6% of patients in the Italian cohort (10), 13% of the Spanish reports (11, 12), and 10% in a study conducted in Zaragoza (23).

AF represents a commonly observed electrocardiographic abnormality among patients with CA, evidenced by its prevalence in various cohorts: 33.7% in the Italian cohort (10), 40% and 53.1% in the studies by Barge-Caballero et al. (11, 12), 15.2% in the Brazilian cohort (14), and 30.8% in our study population. Other described electrocardiographic alterations include low voltage: 30.8% in our study, 35.2% in Italy (10), 42.4% and 27.2% in the Spanish studies (11, 12), 41% in the López-Sainz cohort (13), and 26% in the Zaragoza study (23). Here, an atrioventricular block was found in 30.8%, while higher frequencies are described in both studies by Barge-Caballero et al. (46.3% and 54.3%) (11, 12), and lower frequencies in the Fernandes cohort (20%) (14). This highlights the consistency of electrocardiographic abnormalities in patients with CA across continents.

The main findings in transthoracic echocardiography consistent with CA include unexplained LV thickness, a thickened septum, diastolic dysfunction, and reduced global longitudinal strain (3). These findings were present in most of our patients. Thus, the findings from this study are highly consistent with the established literature on the classic features of CA in echocardiography. Other studies have found dissimilar results such as a higher mean septum thickness compared to our cohort: 16.8 and 16.7 mm in two Spanish cohorts, and 16 mm in an Italian research (10, 11, 13). On the other hand, a Brazilian paper described a median septum thickness of 12 mm in ATTRv and 15 mm in ATTRwt (14). Myocardial speckling was found in 22.5% and 26.5% of Spanish patients, a frequency alike to the one found in our cohort (11).

Interestingly, we found a lower LVEF than in other studies: 62.5% of patients with ATTR had a reduced LVEF and none in the AL group. CA has been described in up to 12% of patients with preserved LVEF and 10% with reduced or mildly reduced LVEF (20). López-Sainz et al. found a mean LVEF of 54.9% in all patients (13), while Barge-Caballero et al. found a LVEF <50% in only 11% of AL cases and 27.6% of ATTR patients (11). In a more recent study by Barge-Caballero et al., these proportions increased to 33.3% and 41.4%, respectively, still lower than those seen in our report (12). Fernandes found a median LVEF of 60.5% in ATTRv, 58.5% in ATTRwt, and 62% in AL CA (14). The Trieste CA registry found a mean LVEF of 55% and 29.5% of the study population had a reduced ejection fraction (10).

Using CMRI, patients with ATTR exhibited a thicker interventricular septum and a higher mass index compared to patients with AL. Similar data were reported by Barge-Caballero et al., where a statistically significant difference was identified between the types of cardiac amyloidosis in this variable (11). Comparatively with a study in the UK including 46 patients with AL and 51 with ATTR, our cohort presented a greater atrial volume, being 50.7 cm^2^ in AL and 61.4 cm^2^ in ATTR compared to 23 and 26 cm^2^ respectively (24). A late gadolinium enhancement pattern was observed in all patients in our cohort with available CMRI. Fernandes et al. reported that 68% of patients presented this finding (14). Barge-Caballero et al. in their Spanish cohort obtained CMRI data in 45 out of 105 patients, demonstrating late gadolinium enhancement in 85.7% of patients with ATTR vs. 58.8% of patients with AL (11).

T1 mapping complements myocardial enhancement and is often found in the early stages of CA before the development of biventricular thickening (25). In our cohort, elevated T1 mapping was present in all patients with CMRI. Moreover, increased extracellular volume has shown a correlation with disease severity. Not all Spanish and Latin American cohorts have this data, because this evaluation is not routinely performed. Our results serve as a reference for future Iberic and American cohorts. Although most radiological findings are consistent across studies, the differences could be highly related to distinct patient characteristics, for instance, a high prevalence of coronary artery disease in certain populations.

## 5 Strenghts and limitations

This study has significant regional importance, as it is one of the first in Colombia to report a case series of patients with CA, highlighted by a comprehensive clinical analysis encompassing demographics, comorbidities, laboratory results, and imaging. The genetic sequencing conducted on all patients provides a deeper insight into the mutations responsible for ATTR-CA within our population, offering substantial clinical and prognostic value, as previously discussed.

Important limitations of this research include the collection of data retrospectively and the exclusion of patients evaluated for CA who were either unreachable or deceased. Furthermore, the inclusion of patients presenting with other causes of cardiac structural changes, such as ischemic cardiomyopathy, may obscure the evaluation of a pure phenotype of CA. Nonetheless, it facilitates a more realistic assessment of patients in clinical settings where such comorbidities are commonly observed. The small sample size presents a limitation, as minor variations in absolute values can substantially impact the reported prevalence. Challenges were also encountered due to missing data related to laboratory tests and imaging, as some were not conducted at our center and no report was obtained.

## 6 Conclusion

The most common causes of CA in our study were AL, ATTRv, and ATTRwt, which predominantly affected older men. Our study's population demographic, laboratory, and radiological characteristics are similar to the literature, which may strengthen the careful application of international studies to patients from our region. Significantly, the p.Val142Ile mutation stands out as the most prevalent pathogenic variant driving ATTRv in our region, a finding of particular concern due to its association with more severe cardiac complications and poorer outcomes. This finding may reflect the substantial African genetic influence in our region.

## Data Availability

The raw data supporting the conclusions of this article will be made available by the corresponding author upon reasonable request.
