# Supplementary figures and images for: A case series of patients with cardiac amyloidosis evaluated at a Colombian university hospital
Source: Front Cardiovasc Med. 2025 Feb 3;12:1487717. doi: 10.3389/fcvm.2025.1487717 (PMC11841452; doi:10.3389/fcvm.2025.1487717)

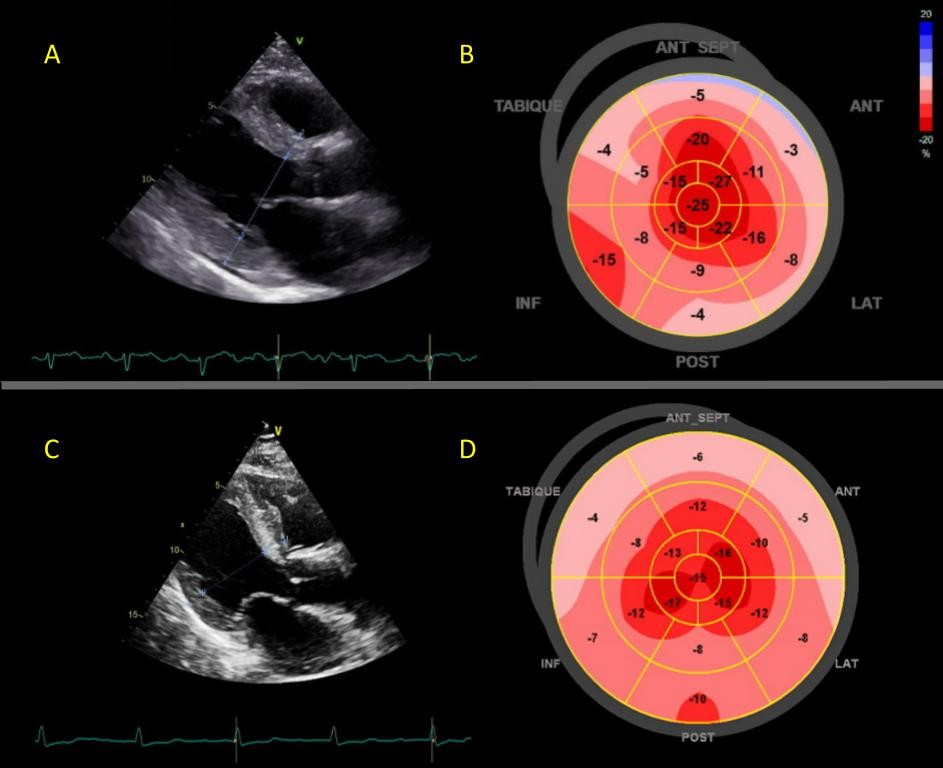

Supplement: Supplementary file 1 [file Image1.jpeg]

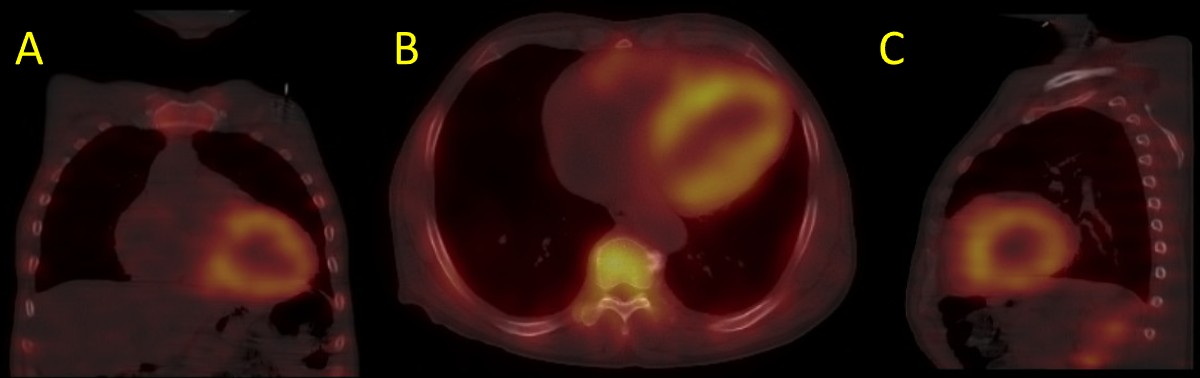

Supplement: Supplementary file 2 [file Image2.jpeg]

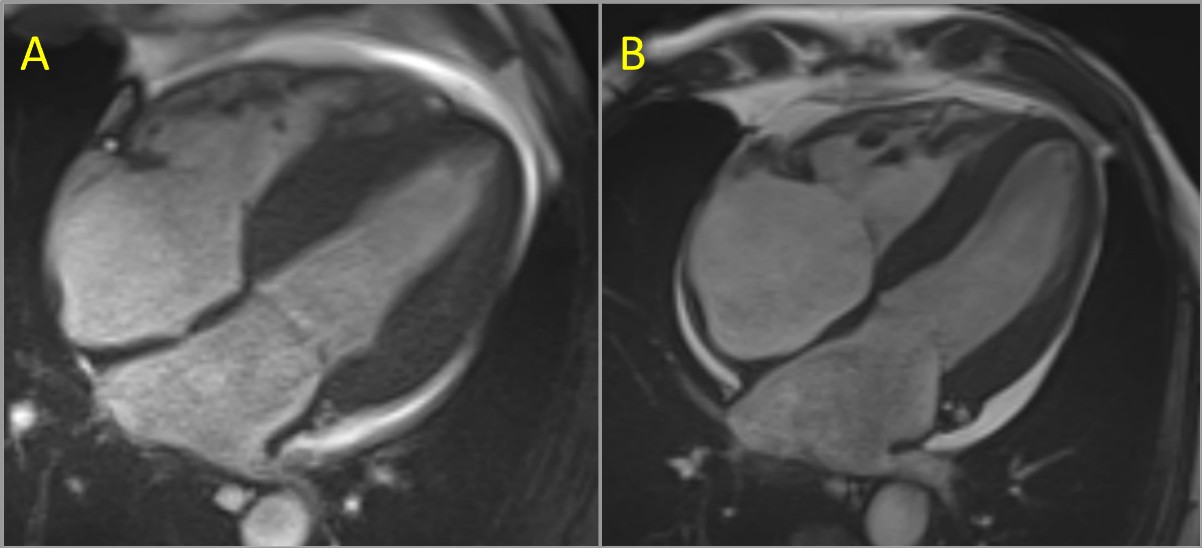

Supplement: Supplementary file 3 [file Image3.jpeg]
